# Editorial: Novel therapeutic target and drug discovery for neurological diseases, volume II

**DOI:** 10.3389/fphar.2025.1566950

**Published:** 2025-02-27

**Authors:** Kaiyue Zhao, Zixuan Li, Ting Sun, Qingshan Liu, Yong Cheng, George Barreto, Zhuorong Li, Rui Liu

**Affiliations:** ^1^ Institute of Medicinal Biotechnology, Chinese Academy of Medical Sciences and Peking Union Medical College, Beijing, China; ^2^ College of Pharmacy, Minzu University of China, Beijing, China; ^3^ College of Life and Environmental Sciences, Minzu University of China, Beijing, China; ^4^ Department of Biological Sciences, University of Limerick, Limerick, Ireland

**Keywords:** Alzheimer’s disease, dementia, depression, drug target, natural products, neurological disorders, neurotrophic factor, therapeutic agents

The research topic “*New Therapeutic Target and Drug Discovery for Neurological Disorders, Volume II*” encompasses 32 articles contributed by 236 authors. This comprehensive collection presents a wide range of content, comprising 13 original research articles, 10 review articles, three systematic reviews, one mini-review, one opinion, one clinical trial, one case report, one brief research report, and one study protocol. The overarching goal of this research topic is to enhance our comprehension of prospective therapeutic targets and the fundamental mechanisms underlying innovative molecular therapies for neurological diseases, ultimately aiming to expedite the advancement of novel treatment approaches.

A Research Topic of articles concentrates on the pathological mechanisms and potential drug targets in neurological diseases. Neuronal death pathways, including ferroptosis, apoptosis, pyroptosis, necroptosis, and autophagy, are critical components in the development of neurological diseases (Moujalled et al., 2021). Jin et al. highlight the importance of pharmacological inhibition of ferroptosis as a novel therapeutic strategy for epilepsy, grounded in an exhaustive examination of the central pathological role and molecular mechanisms of ferroptosis. Wang et al. spotlight the maintenance of endoplasmic reticulum stress to reduce the death of dopaminergic neurons in Parkinson’s disease (PD) through the modulation of key molecules such as protein kinase RNA-like ER kinase (PERK), activating transcription factor 4 (ATF4), endoplasmic reticulum to nucleus signaling 1 (IRE1), and activating transcription factor 6 (ATF6). The study conducted by Nakamura et al. identified pathological alterations in the axon terminals of globus pallidus internus (GPi), characterized by hypertrophy and increased gamma-aminobutyric acid (GABA) release, caused by therapeutic levodopa treatment. This work suggests that gabaergic neuromodulation could be a potential target for levodopa-induced dyskinesia. Xu et al. systematically elaborated the neuroprotective mechanisms of the Apelin/Apelin-receptor (APJ) system in mitigating oxidative stress during stroke, highlighting its promise as a therapeutic target. Beyond neurons, glial cells have received increasing attention in the pathogenesis of neurological diseases. Li et al. utilized a chronic restraint stress (CRS) mouse model to explore how targeting microglia could combat depression. They found that reducing Discs large homolog 1 (Dlg1) expression significantly decreased the number of activated microglia, leading to an improvement in depression-like behavior. In another study, Li et al. demonstrated the role of astrocytes in CRS-induced depression in mice, indicating that enhancing astrocyte energy metabolism and mitochondrial oxidative phosphorylation contributes to the rapid antidepressant effect of hypidone hydrochloride. Zhang et al. explored the role of nuclear factor erythroid 2-related factor 2 (Nrf2)- antioxidant response element (ARE) and signal transducer and activator of transcription 3 (STAT3)- inhibitor of nuclear factor kappa B (NF-κB) zeta (IκBζ) signaling pathways in the treatment of multiple sclerosis with dimethyl fumarate. Moreover, recent studies emphasize the crucial role of epigenetics, particularly noncoding RNAs, in the pathogenesis of neurological diseases (Li et al., 2021; Srinivas et al., 2023; Zeng et al., 2025; Zeng et al., 2021; Zhao et al., 2024). In this subject area, Wang et al. stress the role of microRNA-23b-3p (miR-23b-3p) in Alzheimer’s disease (AD) and its prospects as a therapeutic target. They illustrate that miR-23b-3p alleviates cognitive deficits in APPswe/PSEN1dE9 (APP/PS1) mice by regulating the glycogen synthase kinase-3 beta (GSK-3β)/phosphatase and tensin homolog (PTEN)/phosphorylated tau (p-Tau)/BCL2 associated X-protein (Bax)/Caspase-3 signaling pathways in neurons. Additionally, they have identified small molecules capable of reducing Alzheimer’s systems by acting through the miR-23b-3p-mediated signaling pathways, presenting an inventive therapeutic approach for AD. Another focus is on RNA-binding proteins, such as quaking protein (QKI), which are responsible for noncoding RNA generation and messenger RNA (mRNA) splicing. Guo et al. provide a detailed overview of the role of QKI in the nervous system and suggest its potential as a new target for treating axial gliomas by preserving the stemness of glioblastoma multiforme (GBM) and regulating the tumor microenvironment. Protein modifications, such as palmitoylation, serve as key elements in the pathology of neurological dysfunction, modifying protein function and localization through the addition of palmitate chains (He et al., 2023). Liao et al.thoroughly studied the role of zinc finger DHHC-type (zDHHC) proteins in the brain, proposing that zDHHC and its palmitoylation are potential targets for neurological disorders.

Another group of articles delves into the application of small molecule drugs, peptide drugs, natural products, and traditional Chinese medicine (TCM) in the treatment of nervous system diseases. Feng et al. reported findings on NH300094, a novel small molecule compound, which demonstrated potential efficacy in the treatment of schizophrenia and cognitive impairment due to its 5-hydroxytryptamine receptor 2A (5-HT2A) antagonist activity and antagonism of dopamine D2 and D3 receptors. Meanwhile, two research teams have focused on the role of peptide drugs against neuronal injuries. Gao et al. investigated the therapeutic effects of AV-001 in elderly female rats with vascular dementia (VaD) and multiple microinfarcts (MMI), revealing that this angiopoietin-1 mimicking polypeptide mitigated white matter (WM) damage and improved neurocognitive function. Alternatively, Xiong et al. showed that the parathyroid hormone analogue teriparatide improved the outcome of spinal cord injury (SCI) by reducing blood-spinal cord barrier disruption. Natural products have arisen as pivotal sources for the development of drugs against central nervous system (CNS) disorders (Appendino et al., 2014). Wang et al. illustrated that herbal ingredients exhibited antidepressant effects by protecting neurons via the promotion of neurotrophic factor secretion, including brain-derived neurotrophic factor (BDNF), glial cell-derived neurotrophic factor (GDNF), vascular endothelial growth factor (VEGF), and nerve growth factor (NGF). Lu et al. revealed that *Astragalus polysaccharides* inhibited pentylenetetrazole-induced neuroinflammation in mice, thereby influencing the occurrence of epilepsy and alleviating cognitive impairment. Du et al. demonstrated that Rannasangpei, a traditional Tibetan medicine, and its active component Crocin-1, ameliorated chronic unpredictable mild stress (CUMS)-induced depressive behavior in mice by inhibiting oxidative stress, inflammatory responses, and apoptotic pathways. Dong et al. reported that Jingqianshu granules, containing peony page, Ligusticum essential oil, glycyrrhizin, hesperidin, and paeonol, alleviated premenstrual depression symptoms in rats by modulating the orexin signaling pathway. In addition, the current Research Topic pays particular attention to both the clinical safety and indication expansion of existing drugs. Leu et al. presented a phase I study of intravenous nipocalimab administration in 40 participants, revealing favorable pharmacodynamic and pharmacokinetic properties, suggesting its potential as an effective treatment for immunoglobulin G (IgG)-mediated diseases, such as chronic inflammatory demyelinating polyradiculoneuropathy (CIDP). Zou et al. reported a case of posterior reversible encephalopathy syndrome (PRES) associated with anlotinib and summarized 54 PRES cases caused by antiangiogenic agents, highlighting the critical need for early detection and treatment. Through a comprehensive review of preclinical and clinical studies, Zhang et al. analyzed the challenges associated with ketamine in clinical research, underscoring safety, resistance, and long-term abuse risk as priorities for future research. In a meta-analysis covering 19 articles and 312 studies, Li et al. systematically evaluated the efficacy and safety for the treatment of post-stroke cognitive impairment (PSCI). The findings indicated that therapeutic interventions, including angiotensin-converting enzyme inhibitors, N-methyl-D-aspartate receptor (NMDAR) antagonists, cell therapy, acupuncture, and Western medicine combined with ginkgo biloba extract (EGB761), are capable of significantly improving neurological deficits and activities of daily living, while also demonstrating favorable clinical safety profiles. Drug repurposing is an important strategy in the development of new therapeutics (Ballard et al., 2020). Cui et al.explored the mechanisms by which metformin can be employed for the treatment of dementia and appraised its clinical efficacy, proposing that this medication initiates a pathway for dementia research by improving insulin resistance and providing neuronal protection. Chen et al. proposed a multicenter, double-blind, randomized controlled trial (PSSH) regarding the treatment of spontaneous subarachnoid hemorrhage (SAH) with pioglitazone. Through a meticulously designed study protocol, their research aimed to systematically evaluate the neuroprotective efficacy and safety profile of pioglitazone, offering high-quality clinical evidence to support its potential therapeutic application in SAH.

The final series of articles discusses advanced technologies tailored for the discovery of new drugs and biomarkers in the field of neurological diseases. TCM is a treasure trove for drug development initiatives. Gong et al. used patch-clamp methodologies to screen active compounds in wild guava extract (TSS), uncovering that triterpene saponins C9 and C10 exhibited analgesic effects by regulating transient receptor potential vanilloid 1 (TRPV1) channels in dorsal root ganglion (DRG) neurons. Furthermore, these triterpene saponins suppressed inhibitory synaptic signaling, concurrent with an upregulation of GABA receptors (GABAA) in cortical neurons. Xu et al., through network pharmacological analysis and experimental verification, identified four core components and ten core targets of Erjingwan decoction in the treatment of AD, hinting at the inhibition of the advanced glycation end products (AGEs)/receptor for AGEs (RAGE)/NF-κB signaling pathway as the potential molecular mechanism underlying its efficacy. Fan et al. constructed a fluorescence resonance energy transfer (FRET)-based probe to measure intermediate filament tension and screened a complex containing multiple protein nanoparticles (PNs) and sodium/chloride channel inhibitors by evaluating alterations in intracellular osmotic potential. Their results demonstrated the effectiveness in alleviating astrocyte-induced brain edema toxicity within a rat model of middle cerebral artery occlusion (MCAO). Tang et al. employed integrated metabolomics and bioinformatics analysis techniques to reveal the regulatory effects of Mogroside V (MGV) and its metabolite Mogrol (MG) on 106 metabolites in the substantia nigra of 1-Methyl-4-phenyl-1,2,3,6-tetrahydropyridine (MPTP)-induced PD mice. Their findings demonstrated that MGV and MG significantly modulated key metabolic pathways, including sphingolipid metabolism, fatty acid metabolism, and amino acid metabolism, thereby offering a new theoretical basis for understanding the potential mechanism of *Siraitia grosvenorii* (Swingle) C. Jeffrey in the treatment of PD. Cai et al. focused on the molecular mechanism by which TCM restores disrupted noncoding RNA-mediated signaling pathways to improve mitochondrial function in neurodegenerative diseases, presenting a fresh perspective on the regulation of multi-stage compounds targeting noncoding RNA-associated pathways in TCM. Zhang et al. discussed strategies for intracranial delivery of TCM preparations across the blood-brain barrier, providing a path for the translation of TCM preparations from laboratory research to clinical applications. In the realm of disease biomarker discovery, Bian et al. identified a potential new marker, the tryptophan metabolite indoleacrylic acid, with anti-inflammatory activities through liquid chromatography-tandem mass spectrometry (LC-MS/MS) analysis in the cerebrospinal fluid and serum of patients with neuromyelitis optica spectrum disorders (NMOSD). Chen et al. performed a comprehensive scientometric analysis pertaining to the NMDAR in the context of depression. Their findings revealed that investigating the mechanisms of action of NMDAR antagonists and identifying their molecular targets are pivotal areas of focus in the development of antidepressant therapies. Yang et al. conducted a systematic review and network meta-analysis to compare the efficacy of different cell-derived extracellular vesicles (EVs) in the treatment of traumatic brain injury (TBI), concluding that astrocyte-derived EVs (AEVs) and mesenchymal stem cell-derived EVs (MSC-EVs) are the most promising cell sources for TBI treatment. This finding provides new ideas for the development of TBI therapies.

In summary, this Research Topic covers diverse research endeavors, offering valuable insights into new therapeutic strategies for neurological diseases. Studies centered on epigenetic and neuro-immunological mechanisms have broadened the horizon of potential drug targets for these disorders. Furthermore, cutting-edge technologies such as multi-omics are aiding in the discovery of biomarkers and innovative drug targets. Additionally, elucidating the effects of natural products and TCM, alongside drug repurposing, presents a promising approach for developing effective treatments against intricate neurological conditions. We acknowledge the significant contributions of the editorial team, authors, and reviewers from Frontiers in Pharmacology.
